# Impact of Fungal Hyphae on Growth and Dispersal of Obligate Anaerobic Bacteria in Aerated Habitats

**DOI:** 10.1128/mbio.00769-22

**Published:** 2022-05-31

**Authors:** Bi-Jing Xiong, Sabine Kleinsteuber, Heike Sträuber, Christian Dusny, Hauke Harms, Lukas Y. Wick

**Affiliations:** a Department of Environmental Microbiology, Helmholtz Centre for Environmental Research –UFZ, Leipzig, Germany; b Department of Solar Materials, Helmholtz Centre for Environmental Research –UFZ, Leipzig, Germany; CEH-Oxford

**Keywords:** *Coprinopsis cinerea*, oxygen, hyphae, bacterial-fungal interactions, mycosphere, planar optode, nanoparticles, phase-contrast microscopy

## Abstract

Anoxic microsites arising in fungal biofilms may foster the presence of obligate anaerobes. Here, we analyzed whether and to which degree hyphae of Coprinopsis cinerea thriving in oxic habitats enable the germination, growth, and dispersal of the obligate anaerobic soil bacterium Clostridium acetobutylicum. Time-resolved optical oxygen mapping, microscopy, and metabolite analysis revealed the formation and persistence of anoxic circum hyphal niches, allowing for spore germination, growth, and fermentative activity of the obligate anaerobe in an otherwise inhabitable environment. Hypoxic liquid films containing 80% ± 10% of atmospheric oxygen saturation around single air-exposed hyphae thereby allowed for efficient clostridial dispersal amid spatially separated (>0.5 cm) anoxic sites. Hyphae hence may serve as good networks for the activity and spatial organization of obligate anaerobic bacteria in oxygenated heterogeneous environments such as soil.

## INTRODUCTION

Anoxic and hypoxic microsites foster the presence and activity of even obligate anaerobes in oxic environments (1–4) such as upland soils. As oxygen limitations typically arise if microbial oxygen consumption exceeds diffusive oxygen supply (1, 5), hypoxic microsites often coincide with hot spots of microbial activity such as the rhizosphere (6), the detritusphere (7), or biocrusts (8). Microbial biofilms (9–13) thereby often form steep oxygen gradients with oxygen depletion arising as shallow as ~80 μm beneath the air interface in mycelial layers (14, 15) as detected by needle-type oxygen microsensors (tip size, ~10 μm). Self-induced and spatially confined hypoxic microenvironments also form within filamentous fungal biofilms despite abundant spaces between hyphae, and they are thought to contribute to fungal resistance to antifungal treatments (16). Anoxic fungal niches have also been observed to induce the growth of strict anaerobes (9, 10), suggesting that oxygen depletion may be a specific fungal mechanism to modulate the mycosphere (here defined as areas surrounding and affected by mycelia [17]) chemistry (10, 18). Typically forming 0.05 to 1 mg of biomass dry weight (biomass,dw) per g soil, fungi (19–21) may embody up to 75% of the subsurface microbial biomass (20). Being predominantly aerobic (22), they consume oxygen at rates of up to 180 nM_oxygen_ min^−1^ mg_biomass,dw_^−1^ (14). Unlike in tightly packed industrial biofilms, soil fungi often develop extensively fractal mycelia that allow them to access heterogeneously distributed nutrients and carbon sources (23) and to bridge mycelial source and sink regions (24). Forming hyphae with lengths of ≈10^2^ m g^−1^ in arable and up to 10^4^ m g^−1^ in forest topsoil (20), mycelia thereby also serve as important pathways for bacterial dispersal (“fungal highways”) (25), enabling the colonization of new habitats (26–29), horizontal gene transfer (30), or predation (31). Expressing hydrophobic cell wall proteins (hydrophobins), hyphae thereby overcome air-water interfaces and bridge air-filled pores with nutrient-rich aqueous zones containing little or no oxygen. In upland soils, furthermore, conditions may switch rapidly between oxia and anoxia (1) (e.g., in response to heavy rainfall or waterlogging), leading to a rapidly changing distribution of oxygen. As fungi may both form and bridge anoxic microsites, we here assess to which degree hyphae thriving in oxic habitats enable spore germination, vegetative growth, and dispersal of strictly anaerobic bacteria. Toward this aim, we determined spatial and temporal oxygen profiles around the filamentous mycelia of the fast-growing (~100 μm per h [32]) aerobic coprophilic fungus Coprinopsis cinerea. C. cinerea was chosen as it is a well-characterized, often-used model organism with a known genome (33). It further has been found to grow in soil layers of mown fields or horse/cow dung where anaerobes often co-occur (34) and, hence, is a good representative of a saprobic fungus thriving in upper soil layers. Planar optodes and custom-made micrometer-sized oxygen-sensitive beads determined mycelial oxygen distribution. The spore-forming soil bacterium Clostridium acetobutylicum was chosen as a representative of strict anaerobes due to its inability to grow, ferment, and swim under oxic conditions (35). Its oxygen-tolerant spores only germinate into fully active vegetative cells under anoxic conditions (36). Spatially and temporally resolved oxygen mapping revealed the formation and persistence of anoxic regions in air-exposed C. cinerea mycelia that enabled spore germination and growth of C. acetobutylicum. For the first time, we also document active long-distance (>0.5 cm) dispersal of an obligate anaerobe along air-exposed hyphae between two anoxic sites. Our results demonstrate that the occurrence and activity of aerobic mycelia can create anoxic microniches and facilitate activity and spatial distribution of obligate anaerobes in an otherwise oxic environment. This enlarges our understanding of the ecological role of the mycosphere for microbial dynamics and functional stability at oxic-anoxic interfaces in heterogeneous ecosystems.

## RESULTS

### Time-resolved *in vivo* mycelial oxygen distribution.

**FIG 1:**
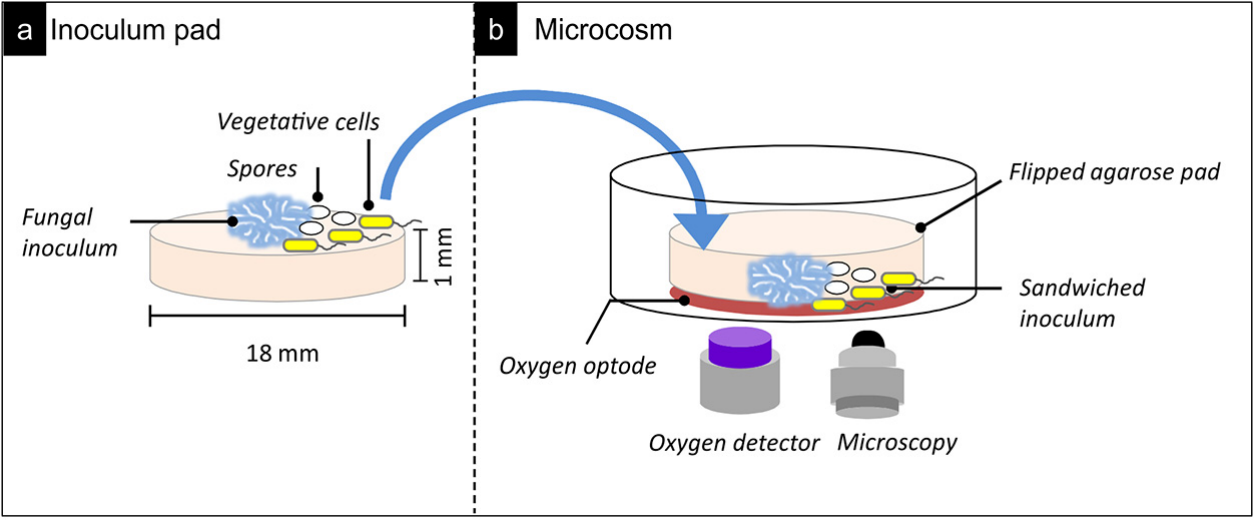
Schematic view of the microcosm used for *in vivo* time-lapse mapping of mycelial oxygen profiles or the detection of mycelia-induced spore germination and bacterial growth. (a and b) An agarose inoculum pad (a) that was inverted and placed in the microcosm (b) to sandwich the fungal inoculum between an oxygen optode at the bottom and the overlying agarose pad. The oxygen optode was glued to the glass bottom of a petri dish, and sensor signals were monitored by a commercial detector. The development of the fungal networks on the optode surface was imaged by bright-field microscopy. In a parallel experiment, vegetative cells or spores were placed at the center of agarose inoculum pad or 3 mm away from the fungal inoculum, respectively. Growth, spore germination, and activity of obligate anaerobe were followed by microscopy.

**FIG 2:**
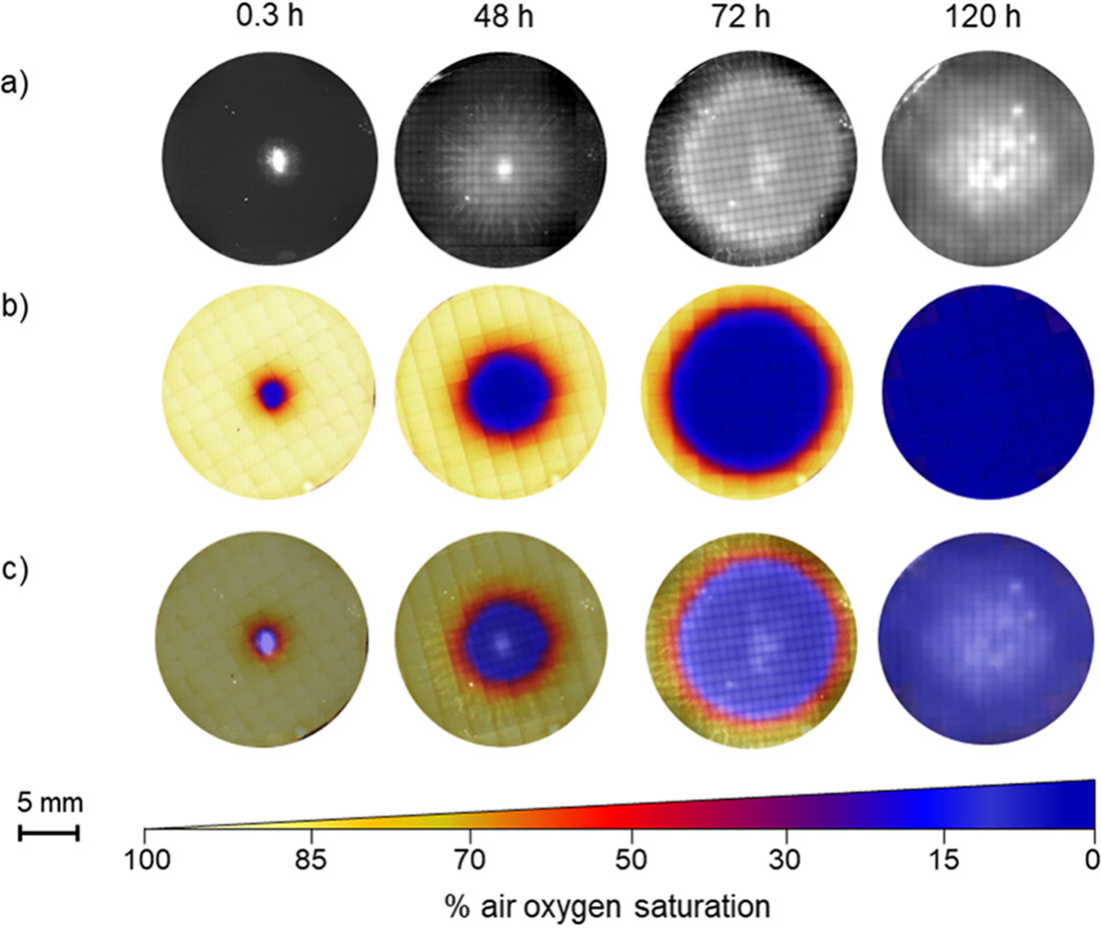
Growth and *in vivo* time-lapse mycelial oxygen profiles of C. cinerea (see Video S1 in the supplemental material). (a and b) Micrographs of the development of mycelial growth and mycelial oxygen profiles of C. cinerea monitored over 120 h at 30°C using an oxygen optode. (c) Overlay of optode and bright-field micrographs. Dark-blue and light-yellow colors refer to ca. 0% to 100% of air oxygen saturation in water at 30°C (see color bar).

Combining microscopic observation and optical oxygen sensing of laboratory microcosms (Fig. 1), we mapped growth and spatiotemporal oxygen concentrations in aerated mycelia of C. cinerea (Fig. 2; see Video S1 in the supplemental material). Zones of oxygen depletion formed at the inoculation point as shortly as <6 min (Video S1) after attaching the pad to the oxygen optode. Diameters of oxygen-free zones then steadily expanded from 2.3 ± 0.8 mm at time (*t*) of 0.3 h to 18 ± 0.0 mm at *t* of 120 h. At the mycelial borders, we observed steep oxygen gradients from 100% to 0% air saturation over a distance of ~2 mm (Fig. 2, row c).

10.1128/mbio.00769-22.8 VIDEO S1 Oxygen optode revealing rapid oxygen consumption by C. cinerea and oxygen depletion around C. cinerea inocula. Download Movie S1, AVI file, 10.4 MB. Copyright © 2022 Xiong et al. 2022 Xiong et al. https://creativecommons.org/licenses/by/4.0/ This content is distributed under the terms of the Creative Commons Attribution 4.0 International license.

### Growth and fermentative activity of C. acetobutylicum in the presence of mycelia.

To validate the presence of the optically mapped oxygen-free zones, we inoculated C. acetobutylicum cells to C. cinerea and tested if mycelial activity enables growth of the obligate anaerobe. After 7 days of air-exposed growth in the presence of mycelia, C. acetobutylicum formed dense colonies in the mycosphere (Fig. S1a; *t* = 7 days), accounting for a >1,300-fold increase in cells numbers from 3.5 × 10^5^ ± 3.3 × 10^3^ (*t* = 0 days) to 4.8 × 10^8^ ± 3.5 × 10^7^ cells g^−1^_agar_ at *t* of 7 days (Fig. S1c). Micrographs revealed typical traits of C. acetobutylicum, including rod-shaped cells and the formation of optically brighter regions at one pole (Fig. S1a; *t* = 7 days) as signs of endospore formation. Comparison of total and viable cell counts (Fig. S1c) further showed that >75% of the total cells were viable at *t* of 7 days. DNA extraction and subsequent 16S rRNA gene sequencing confirmed that C. acetobutylicum was the only bacterium present in the microcosms, as the partial sequence was identical to the published 16S rRNA gene of C. acetobutylicum (GenBank accession no. NR_074511). In the absence of mycelia, no growth of the obligate anaerobes was observed (Fig. S1b) with cell numbers that were too low to be detected by the cell counter (Fig. S1c). Bacterial growth in the presence of mycelia went along with the production of significant amounts of butyrate and 1-butanol, i.e., typical water-soluble products of clostridial fermentative metabolic activity (Fig. S2; Text S1). No such metabolites were found in controls with only C. acetobutylicum or C. cinerea inocula under aerobic conditions.

10.1128/mbio.00769-22.1 TEXT S1 Details on supplemental Materials and Methods. Download Text S1, DOCX file, 0.04 MB. Copyright © 2022 Xiong et al. 2022 Xiong et al. https://creativecommons.org/licenses/by/4.0/ This content is distributed under the terms of the Creative Commons Attribution 4.0 International license.

10.1128/mbio.00769-22.2 FIG S1 C. acetobutylicum cell development after 7 days in the presence and absence of hyphae of C. cinerea. (a and b) Micrograph of C. acetobutylicum cells after 7 days in the presence (a) and absence (b) of C. cinerea. (c) C. acetobutylicum cell number density on the day of inoculation (*t* = 1 day) and on the day of harvest (*t* = 7 days) in the presence and absence of the fungus C. cinerea. Arrows highlight suspected polar attachment of C. acetobutylicum to C. cinerea hyphae. Data represent the average and standard deviation (*n *= 3) of total and viable C. acetobutylicum cell counts. ND, not detected. Download FIG S1, TIF file, 2.3 MB. Copyright © 2022 Xiong et al. 2022 Xiong et al. https://creativecommons.org/licenses/by/4.0/ This content is distributed under the terms of the Creative Commons Attribution 4.0 International license.

10.1128/mbio.00769-22.3 FIG S2 Concentration of butyrate and 1-butanol in agarose pads with different inocula. Butyrate and 1-butanol were solely detected in agarose pads inoculated with C. acetobutylicum and C. cinerea growing under ambient oxic conditions. Data represent mean and standard deviation of *n *= 2 for agar pads inoculated with C. acetobutylicum and C. cinerea and *n *= 3 for the treatments with C. acetobutylicum only, C. cinerea only, or agar only. BDL, below the detection limit. Download FIG S2, TIF file, 0.3 MB. Copyright © 2022 Xiong et al. 2022 Xiong et al. https://creativecommons.org/licenses/by/4.0/ This content is distributed under the terms of the Creative Commons Attribution 4.0 International license.

### Hypha-induced germination of C. acetobutylicum spores.

**FIG 3:**
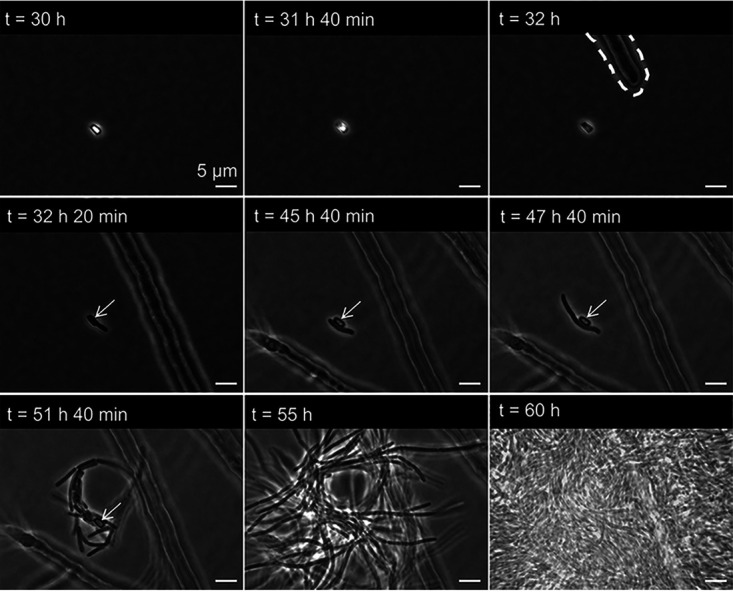
Germination and growth of C. acetobutylicum spores in the mycosphere of C. cinerea. At *t* of 30 h, phase-bright appearance of a dormant spore initially inoculated ca. 3 mm from the hyphal tip. At *t* of 31 h 40 min, swelling of spore; one pole of the spore is less bright. At *t* of 32 h, appearance of hyphae and spore without phase-bright appearance. At *t* of 32 h 20 min, appearance of germ tube. At *t* of 45 h 40 min, longitudinal growth phase is completed. At *t* of 45 h 40 min to 60 h, symmetric cell division and formation of bacterial colony. White arrows point at empty spore shells after germination.

Microscopic observation of C. acetobutylicum germination near active growing C. cinerea hyphae further evidenced the presence of anoxic niches in the mycosphere (Fig. 3; Video S2). Time-lapse microscopic imaging revealed a phase-bright appearance of dormant spores (Fig. S3; Text S1) in the absence of hyphae at *t* of 0 to 30 h (Video S2). At *t* of 30 to 32 h (Video S2), the halo surrounding of the spores gradually increased, and spores lost their phase-bright appearance between *t* of 31 h 40 min and *t* of 32 h, i.e., when the first hyphal tip became visible at a distance of 13 μm from the spore (Fig. 3). At *t* of 32 h 20 min, the shedding of the spore finished and the formation of a dense bacterial mat became visible (*t*, 32 h 20 min to 60 h; Fig. 3). The mat was demonstrated to consist of C. acetobutylicum by 16S rRNA gene sequencing. In the absence of hyphae, no clostridial spores germinated (Fig. S4).

10.1128/mbio.00769-22.4 FIG S3 Phase-contrast micrograph of C. acetobutylicum spores. White dashed arrows point at spores released from mother cells. White solid arrows highlight endospores in the swollen, cigar-like mother cells. Download FIG S3, TIF file, 2.6 MB. Copyright © 2022 Xiong et al. 2022 Xiong et al. https://creativecommons.org/licenses/by/4.0/ This content is distributed under the terms of the Creative Commons Attribution 4.0 International license.

10.1128/mbio.00769-22.5 FIG S4 C. acetobutylicum spores in the absence of C. cinerea mycelia exposed to oxic condition over 60 h of incubation. Download FIG S4, TIF file, 0.7 MB. Copyright © 2022 Xiong et al. 2022 Xiong et al. https://creativecommons.org/licenses/by/4.0/ This content is distributed under the terms of the Creative Commons Attribution 4.0 International license.

10.1128/mbio.00769-22.9 VIDEO S2 Germination and growth of C. acetobutylicum spores in the mycosphere of C. cinerea. Download Movie S2, AVI file, 12.3 MB. Copyright © 2022 Xiong et al. 2022 Xiong et al. https://creativecommons.org/licenses/by/4.0/ This content is distributed under the terms of the Creative Commons Attribution 4.0 International license.

### Dispersal of C. acetobutylicum along air-exposed mycelia.

**FIG 4:**
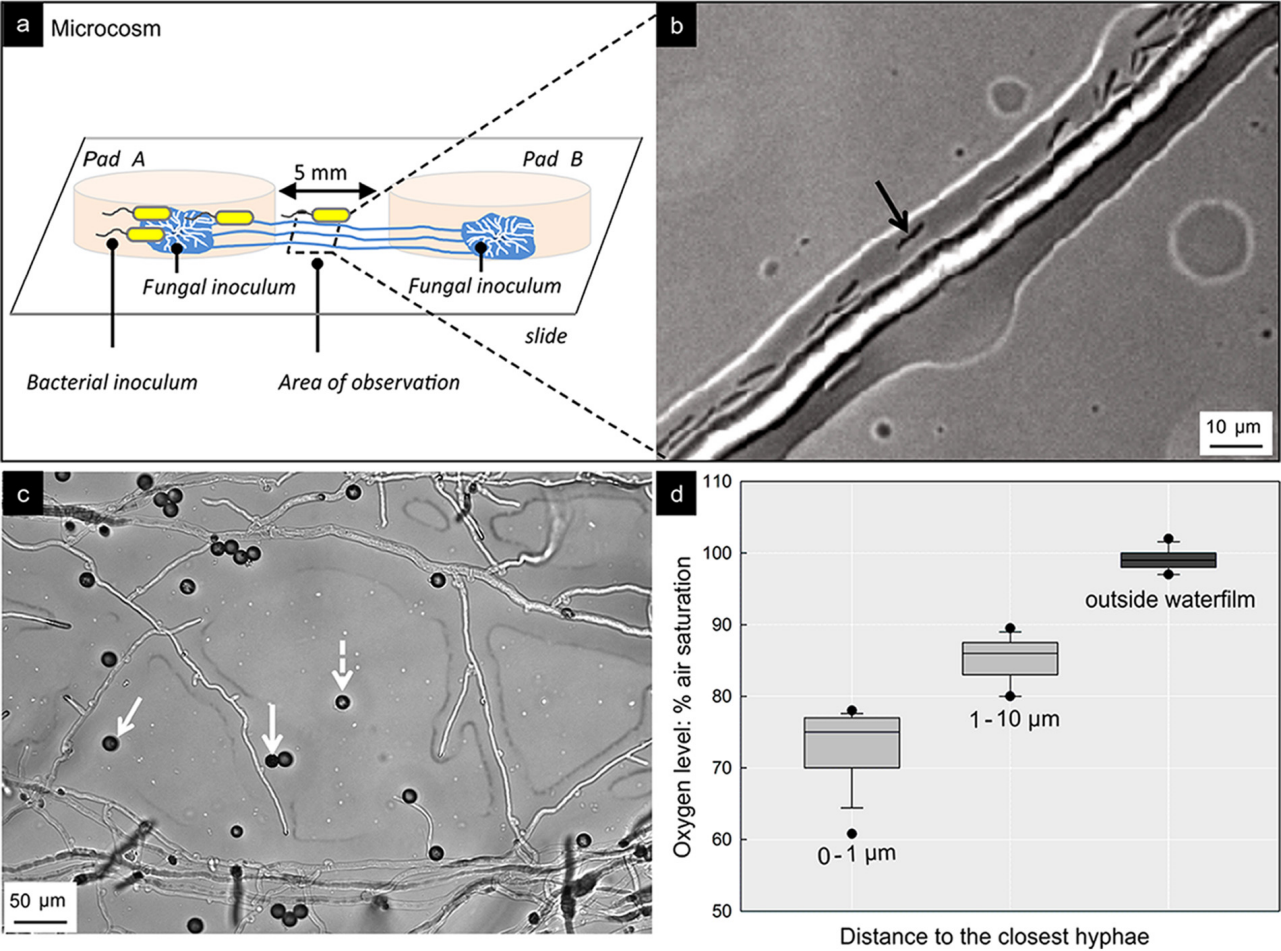
Spatial dispersal of C. acetobutylicum along C. cinerea hyphae. (a) Schematic view of the microcosm used for time-lapse monitoring of dispersal of C. acetobutylicum cells along air-exposed hyphae of C. cinerea. Agar pad A was inoculated with C. cinerea and C. acetobutylicum and pad B with C. cinerea only. (b) Micrograph of C. acetobutylicum swimming (cf. black arrow) along air-exposed hyphae of C. cinerea between agar pads A and B at *t* of 5 days. (c) Micrograph of representative distribution of oxygen-sensitive beads (Ø, 8 μm) between hyphae of C. cinerea overgrowing an agar surface. White arrows exemplify the presence of beads immersed in the hyphal liquid film extending to ca. 10 μm around hyphae. (d) Distribution of sensed oxygen concentration by oxygen-sensitive beads in apparent distance from hyphal surface (80% ± 7.4% air oxygen saturation, 0 to 10 μm, *n *= 53; 99% ± 1.5% air oxygen saturation, >10 μm, *n *= 13).

C. acetobutylicum cells growing in the mycosphere of C. cinerea in the agar pad A (Fig. 4a) were observed remaining motile and started to colonize and disperse along hyphae, linking the two nutrient-rich agarose pads (Fig. 4a) at *t* of 5 days (Fig. 4b; Video S3). Such dispersal resulted in the colonization of agar pad B (Fig. 4a) and subsequent growth of C. acetobutylicum therein (Fig. S5). In the absence of hyphae, no clostridia between the two agar pads and no cells in agar pad B were observed. In a separate experiment, we further analyzed the effect of oxygen on the swimming of C. acetobutylicum. Micrometer-sized lifetime-based oxygen-sensitive beads near air-exposed hyphae (Fig. 4c) revealed that oxygen levels decreased to 73% ± 5% air saturation (Fig. 4d; 0 to 1 μm) at the direct hyphal surface, 85% ± 3% in the apparent liquid film at distances of 1 to 10 μm above the hyphal surface (Fig. 4d; 1 to 10 μm), and 100% ± 1% outside the hyphal liquid film (Fig. 4d). To study the effect of oxygen on clostridial motility, we microscopically assessed the dispersal of C. acetobutylicum cells in liquid medium with 80% and 100% air-saturated oxygen concentrations: 2.2%, 1.2%, and 0.8% of the observed cells actively swam after 1, 15, and 30 min, respectively, in ~80% air saturated media (Table S1). No active swimming was observed, however, in a fully air-saturated environment (Table S1).

10.1128/mbio.00769-22.6 FIG S5 Extracted DNA after 7 days in agar pads A and B in presence of C. acetobutylicum and C. cinerea (i), C. acetobutylicum (ii), and C. cinerea (iii). Low DNA amounts (<1 μg g^−1^) in controls with C. cinerea only pointing at a low DNA extraction efficiency for C. cinerea by the method applied. DNA amounts presented in the figure are hence likely to predominantly reflect extracted DNA from C. acetobutylicum. Data represent the average and standard deviation of *n *= 3 DNA extractions. Download FIG S5, TIF file, 0.2 MB. Copyright © 2022 Xiong et al. 2022 Xiong et al. https://creativecommons.org/licenses/by/4.0/ This content is distributed under the terms of the Creative Commons Attribution 4.0 International license.

10.1128/mbio.00769-22.7 TABLE S1 Fraction of actively swimming **C. acetobutylicum** cells in liquid SM824 medium at different oxygen levels. The fraction refers to the percentage of *n* = 249 to 281 cells detected in the focal plane during microscopic observation of triplicate samples. Download Table S1, PDF file, 0.4 MB. Copyright © 2022 Xiong et al. 2022 Xiong et al. https://creativecommons.org/licenses/by/4.0/ This content is distributed under the terms of the Creative Commons Attribution 4.0 International license.

10.1128/mbio.00769-22.10 VIDEO S3 C. acetobutylicum swimming along C. cinerea air-exposed hyphae. Download Movie S3, AVI file, 11.4 MB. Copyright © 2022 Xiong et al. 2022 Xiong et al. https://creativecommons.org/licenses/by/4.0/ This content is distributed under the terms of the Creative Commons Attribution 4.0 International license.

## DISCUSSION

### Mycelia form anoxic niches and enable activity of anaerobes.

By combining oxygen-sensing and culture-based approaches, our study shows that growing mycelia of C. cinerea form anoxic niches (Fig. 2), allowing for development (Fig. 3; see Fig. S1 in the supplemental material) and metabolic activity (Fig. S2) of the obligate anaerobic bacterium C. acetobutylicum. Although few studies report anoxic niches in fungal biofilms (10, 16, 18), effects of fungal oxygen consumption on bacterial-fungal interactions are still poorly described. Biofilms of oxygen consumption by the candida yeasts created anaerobic niches in the oral cavity (18), thereby influencing the composition of its associated microbiome by favoring anaerobic bacteria over aerobes (27, 37). Being prevalent in heterogeneous terrestrial habitats, fungi often form dense mycelial mats on dead organic material or exist in fungal-bacterial biofilms. Metabolically active fungi are thereby likely to create spatially distinct anoxic/hypoxic niches. Assuming typical fungal oxygen consumption of 1.63 × 10^−3 ^mol m^−3^ s^−1^ (15, 38, 39), a rough calculation (for details, see Text S1) indicates that ~543 μg g^−1^_soil_ of fungal biomass would suffice to create anoxia if the mycelia were covered by a water film of 1 mm thickness. Given a typical fungal biomass density of 50 to 1,000 μg g^−1^_soil_ (19–21), our calculation suggests that aerobic fungi may play an overlooked role in creating (and bridging; see below) anoxic microniches in otherwise oxic habitats. Such an assumption is in line with the observed formation of anoxic habitats at the periphery of oxygen-consuming fungal-bacterial hot spots in soil (5, 6, 40). It also supports the previously described co-occurrence of aerobes and anaerobes within microbial communities in aerated soils (4, 41) and fecal microbiomes (34), or the presence of genes responsible for the anaerobic biosynthetic pathways in the mycosphere (7, 8).

Figure S1a suggests that some C. acetobutylicum cells may attach to and cluster along hyphae of C. cinerea in an end-on manner, pointing at possible antagonistic interactions, as has been described for polar attachment of Bacillus subtilis NCIB 3610 to C. cinerea (42) or of Paenibacillus polymyxa to Fusarium oxysporum (43), respectively. In return, to defend against bacterial attack and to compete within ecological niches (44), C. cinerea is known to produce antibacterial peptides and proteins, such as the cysteine-stabilized αβ defensin copsin (45). No apparent negative interaction of C. cinerea with C. acetobutylicum, however, was observed during clostridial dispersal and germination in the mycosphere (Fig. S1 and S2; Video S2).

### Mycelia mediate activity and spatial organization of anaerobes in air-exposed habitats.

While the mycosphere is known as a typical habitat for aerobic bacteria (46, 47), our study reveals that hyphae may also evoke the germination, activity, and growth of spores of anaerobic C. acetobutylicum in an aerated habitat (Fig. 3; Video S2). Spore germination of C. acetobutylicum thereby started already at a distance of ~13 μm from the hyphal tip (Fig. 3). This suggests that hyphal oxygen consumption affected similar regions as described for pH changes (32), enzymatic activity (48), and the dispersal of bacteria (30, 49). Air-exposed hyphae of C. cinerea by this means also enabled the dispersal of C. acetobutylicum and the colonization of anoxic habitats separated by ambient air (Fig. 4; Video S3).

Even though fermentation and growth of C. acetobutylicum are immediately halted by the presence of oxygen (35), literature shows that C. acetobutylicum (50) and other strict anaerobes adapt to oxygen exposure by using defense mechanisms to minimize oxygen stress (2, 51). Our experiments (Table S1), for instance, revealed that ca. 1% of C. acetobutylicum cells remained up to 30 min motile if exposed to 80% air-saturated medium (Table S1), i.e., oxygen levels similar to those observed by oxygen-sensing microbeads in a liquid film formed around hyphae (Fig. 4d). As clostridia characteristically express the highest motility in the exponential growth phase (52), the most active cells may thus move along hypoxic hyphae. Assuming a mean swimming speed of velocity (*v*) of *5* to 25 μm s^−1^ (53, 54) and active swimming for *t* of ~30 min, mycosphere clostridia would be able to disperse over distances of *s *=* v *×* t* of 27 to 45 mm between anoxic niches.

**FIG 5:**
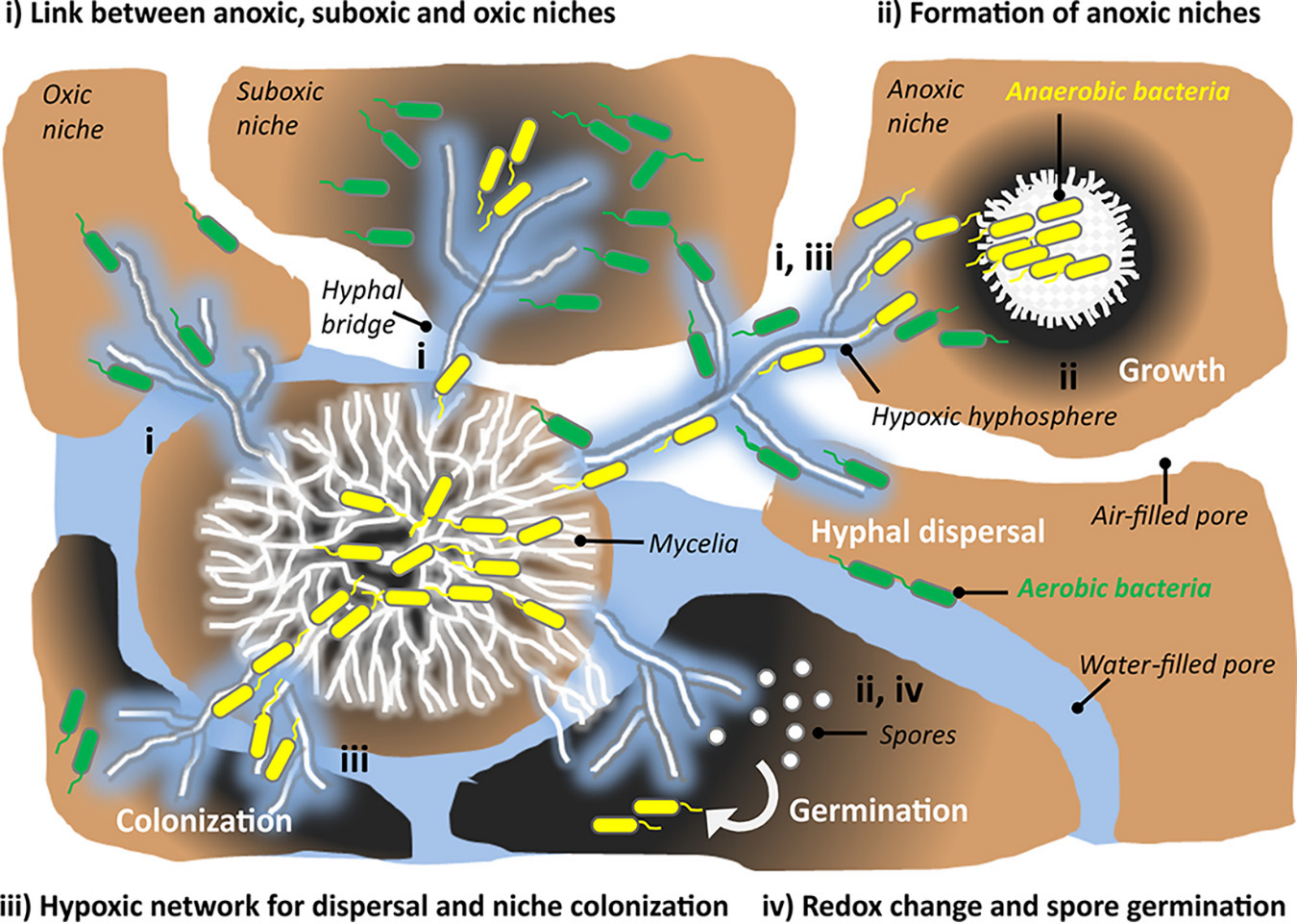
Mycelia mediate activity and spatial organization of anaerobic microorganisms. By forming extended fractal networks, filamentous fungi are well adapted to heterogeneous soil environments, which often may form microbial hot spots and anoxic/hypoxic microniches. (i) Due to their fractal structure and ability to cope with short-term hypoxic conditions, fungi may form stable networks that form hyphal links between anoxic/hypoxic, oxic, and/or oxic-anoxic niches in soil. (ii) Being predominantly aerobic, fungal mycelia may consume oxygen at higher rates than can be delivered by their environment. This leads to the formation of mycelial anoxic niches that enable growth and activity of anaerobic bacteria. (iii) Active hyphae also reduce the oxygen content in their mycosphere. Even if air exposed, mycelia hence may form a hypoxic network for dispersal of anaerobic and aerobic bacteria that enable the colonization of new niches. (iv) Reduction of mycosphere oxygen content and changes of concomitant redox conditions also stimulate the activity of anaerobes as reflected, for instance, by the germination of their spores. Dark-gray color in the figure refers to reduced oxygen level; yellow and green cells refer to (strictly) anaerobic bacteria and aerobic bacteria, respectively.

Our observation that obligate anaerobe C. acetobutylicum actively swims in the hypoxic liquid around hyphae suggests that mycelia may not only mediate the transfer of aerobes (25) but also anaerobes (Fig. 4) between spatially separated habitats (as summarized in Fig. 5). Such finding seems particularly important for soil, as soil microbial communities are constantly exposed to fluctuating conditions (55, 56) that can lead to physicochemically distinct zones, habitat patchiness, and the formation of hot spots of microbial activity. Localized microbial activity likewise may lead to the formation of oxygen gradients (5) and spatial separation of aerobes and anaerobes (57). Fractally growing fungi in soil (23) thereby may often overgrow and link habitats with different oxygen concentrations. Due to their ability to cope with short-term hypoxic conditions (e.g., by the use of fermentative metabolism [58]), fungal networks may thus stably bridge oxic, oxic-anoxic, or anoxic interfaces (Fig. 5) even under various environmental conditions. Given the short-term tolerance of strict anaerobes to oxygen and presumed reduced oxygen contents in the mycosphere, we propose that hyphae in spatially heterogeneous environments may serve as a good network for the activity and spatial organization of a wide range of bacteria, including obligate anaerobes, facultative anaerobes, aero-tolerant anaerobes, microaerophiles, and aerobes. Exploring mycosphere processes beyond traditionally assumed boundaries will further unravel the interactions, functioning, and stability of fungal-bacterial communities in microbial ecosystems.

## MATERIALS AND METHODS

### Microorganisms, medium, and growth conditions.

The obligate anaerobe C. acetobutylicum DSM 792 (type strain, purchased from DSMZ-German Collection of Microorganisms and Cell Cultures, Braunschweig, Germany) was cultivated for 3 days at 37°C under strictly anoxic conditions with an N_2_ headspace in 200-mL serum bottles containing 50 mL of SM824 medium (59). Using a syringe, 2 mL of the culture with an optical density at 600 nm (OD_600_) of 0.81 was then removed and centrifuged at 4,000 × *g* at 10°C for 5 min, the supernatant was discarded, and the cells were resuspended in 2 mL air-saturated SM824 medium and then used as inoculum (see below). The remaining culture was further cultivated until endospore formation was observed (typically at *t* of 7 days) (see Fig. S3 and Text S1 in the supplemental material). C. acetobutylicum spores were obtained as described by Yang et al. (60) (see Text S1) to form inocula of an OD_600_ of <0.02 in SM824 medium (see below). The basidiomycete C. cinerea strain AmutBmut (61) served as filamentous fungus (33). It was cultivated at 30°C for 3 days on yeast-malt extract-glucose medium (42). Using a scalpel, a small piece (diameter < 1 mm) of C. cinerea inoculum was cut from the peripheral growth zone and used as inoculum in the microcosms.

### Time-lapse *in vivo* mapping of microcosm of mycelial oxygen profiles.

All microcosms were handled and used under laboratory atmosphere conditions. Microcosms for mycelial profile mapping consisted of a fungus-inoculated agarose pad (Ø, 18 mm; height (*h*), 1 mm) (Fig. 1a) that was inversely placed on an oxygen optode (Ø, 18 mm; SF-RPSu4; PreSens, Regensburg, Germany) (Fig. 1b) as described earlier (32). Briefly, 400 μL of aerated SM824 medium (1.5% low-melt agarose; Carl Roth, Karlsruhe, Germany) was placed on a circular cover slide (Ø, 18 mm; ibidi, Gräfelfing, Germany), immediately covered by a second cover slide, and allowed to cool for 10 min. After removing the top slide with tweezers, the agarose pad was centrally inoculated with a small piece (Ø < 1 mm) of fungal inoculum and incubated for 48 h at 30°C. The pad was then flipped over and attached to an oxygen optode that itself was glued to the glass bottom of a petri dish (*μ*-Dish 35 mm, low; ibidi). The second cover slide was removed immediately. Five sterile agarose pads (Ø, 8 mm) cut from a 1-cm-thick SM824 agar plate were evenly placed around the inoculated pad to keep the microcosm moisturized.

### Mycelial growth and oxygen distribution in the mycosphere.

Immediately after placing the pad (*t* = 0 h), oxygen profiles around the fungal inoculum (area, 1.8 by 2.5 mm) were monitored for 15 min at intervals of 30 s, using a commercial detector unit (VisiSens TD detector unit DU02; PreSens). Combining microscopic observation (Nikon AZ100; Amsterdam, The Netherlands), and optical sensing techniques, the mycelial development and oxygen concentrations in the whole pad were then mapped at *t* of 0.3, 48, 72, and 120 h. As the commercial oxygen sensing unit did not support centimeter-scale sensing, the microcosm was fixed to a connection arm (edelkrone, Langen, Germany) that was mounted on a software-controlled microscope stage (NIS-Elements Basic Research; Nikon). To map the agarose pad with an area of ca. 250 mm^2^, >100 spots of 1.8 by 2.5 mm were analyzed and stitched using open-source software ImageJ (https://imagej.nih.gov/ij/). An interval of 3,000 ms was allowed between shifting two spots. In this period, the oxygen profile at a given spot was measured with the detector unit. After oxygen mapping (duration, ~15 min), bright-field microscopic images of the mycelium were taken at each time point using the large image acquisition function of the microscope (×15 magnification). The microcosms (Fig. 1b) were stored at 30°C in the dark between sampling times. Triplicate experiments were performed. The calibration of the oxygen optode is described in the Text S1.

### Hypha-induced vegetative growth and spore germination of C. acetobutylicum.

Similar microcosms as described above (Fig. 1b), yet without the semitransparent oxygen sensor, served to examine the vegetative growth and fermentative activity of C. acetobutylicum in the presence of C. cinerea. One microliter of a C. acetobutylicum suspension (OD_600_ of 0.81, or ca. 8.1 × 10^7^ cells mL^−1^) was inoculated to the center of the agar pad that had been preincubated with C. cinerea for 48 h. Then, the pad was flipped over and attached to the glass bottom of a Petri dish (μ-dish 35 mm, low; ibidi). To moisturize the air in the petri dish, five sterile agarose pads (Ø, 8 mm; *h*, 1 cm) were evenly placed around the inoculated pad. The microcosms were incubated as described above and examined daily with a microscope. At *t* of 7 days, the microcosms were harvested for (i) DNA extraction and Sanger sequencing of the 16S rRNA gene, (ii) total and viable bacterial cell counting, and (iii) analysis of water-soluble metabolites (see Text S1). Sterile pads and pads inoculated with C. cinerea only or C. acetobutylicum only served as controls. All experiments were performed in triplicate. DNA was extracted using a commercial extraction kit (DNeasy PowerSoil kit; Qiagen, Hilden, Germany) following the manufacturer’s protocol and quantified with a NanoDrop 1000 spectrophotometer (Thermo Fisher Scientific Inc., Waltham, MA, USA). The 16S rRNA gene was partially amplified and sequenced on an ABI Prism 3130xl genetic analyzer (Applied Biosystems GmbH, Weiterstadt, Germany) using the primers of 27f and 519r as described elsewhere (62). For total and viable cell counting, bacterial cells in the microcosms were recovered as described in Text S1. Total and viable bacterial cell counting was done using an automated microbial cell counter (Quantom Tx; Logos, Gyeonggi, South Korea) with a commercially available total and viable cell staining kit (Logos Biosystems, Suwon, Gyeonggi-do, South Korea), respectively.

For spore germination experiments, 3 μL of spore suspension (OD_600_ < 0.02) was inoculated to the agarose pad (Fig. 1a) at a distance of ~3 mm from the fungal inoculum, and the microcosm was incubated in the dark for 24 h at 30°C under laboratory atmosphere conditions. After 24 h, spore germination and the subsequent cellular growth were monitored by phase-contrast microscopy (exposure time, 300 ms; LED light source intensity, 4.7 V) at 20-min intervals for 36 h. During the microscopic imaging, the microcosm was incubated at constant temperature (30°C) by using a heating system (XLmulti S2; Carl Zeiss Microscopy GmbH, Jena, Germany) mounted to the microscope (Axio Observer; Carl Zeiss Microscopy GmbH). At the end of the experiments, the microcosms were harvested for DNA extraction and 16S rRNA gene sequencing as described above.

### Dispersal of C. acetobutylicum along hyphae.

Two 1-mm-thick agarose pads were inoculated with C. cinerea (see above; Fig. 1a) and incubated at 30°C for 48 h. After that, 1 μL of vegetative C. acetobutylicum cells (OD_600_, 0.81) was placed onto the fungal inoculum in one agarose pad. Immediately thereafter, the fungus-bacteria-inoculated pad (agar pad A) and exclusively fungus-inoculated pad (agar pad B) were both flipped over and placed at a distance of 5 mm on a microscopy slide (26 by 76 mm; ibidi). The microscopy slide was then transferred to a plastic petri dish (90 mm; Thermo Fisher, Waltham, USA), and five circular agarose pads (Ø, 10 mm; *h*, 1 cm; SM824 agar) were evenly placed around the slide to keep the microcosm moisturized during incubation for 7 days at 30°C and ambient air. All microcosms were prepared in triplicate and monitored daily by microscopy to examine the formation of hyphae between two agar pads and the presence of C. acetobutylicum cells moving along them, respectively. To exclude potential bacterial contamination and to provide quantitative molecular evidence for the presence of C. acetobutylicum, DNA was extracted, and DNA concentration was measured photometrically using a NanoDrop ND-1000 UV-visible (UV-Vis) spectral photometer (Thermo Fisher Scientific Inc.) from both agar pads at *t* of 7 days. DNA was sequenced as described above.

### Hyphal oxygen mapping by lifetime-based oxygen-sensitive beads.

Oxygen content in the liquid film surrounding C. cinerea hyphae was also measured using custom-made, lifetime-based, oxygen-sensitive beads (Ø = 8 μm). C. cinerea was inoculated to the 1-mm-thick agarose pad and incubated at 30°C for 48 h. The oxygen-sensitive beads were dispersed in sterile deionized water, and three drops (1 μL each drop) of suspension were placed at ~2 mm distance from growing hyphal tips to the agar surface. The agarose pad was then incubated overnight at 30°C to let the hyphae overgrow the beads. Position- and lifetime-based luminescence quenching of the ruthenium-phenanthroline-based phosphorescence dye in individual oxygen beads was measured using an Opal system (Colibri Photonics; ibidi) connected to an automated inverted Zeiss microscope (Axio Observer, Carl Zeiss Microscopy GmbH). The 532 nm LED of the Opal and a filter cube with 531/40 nm (excitation) and 607/70 nm (emission) were inserted in the optical light path during measurements. Text S1 describes the calibration of the oxygen beads.

Swimming activity of C. acetobutylicum in liquid SM824 medium with different oxygen levels was examined microscopically as described in Text S1.
