# Supplementary figures and images for: Impact of Fungal Hyphae on Growth and Dispersal of Obligate Anaerobic Bacteria in Aerated Habitats
Source: mBio. 2022 May 31;13(3):e00769-22. doi: 10.1128/mbio.00769-22 (PMC9239063; doi:10.1128/mbio.00769-22)

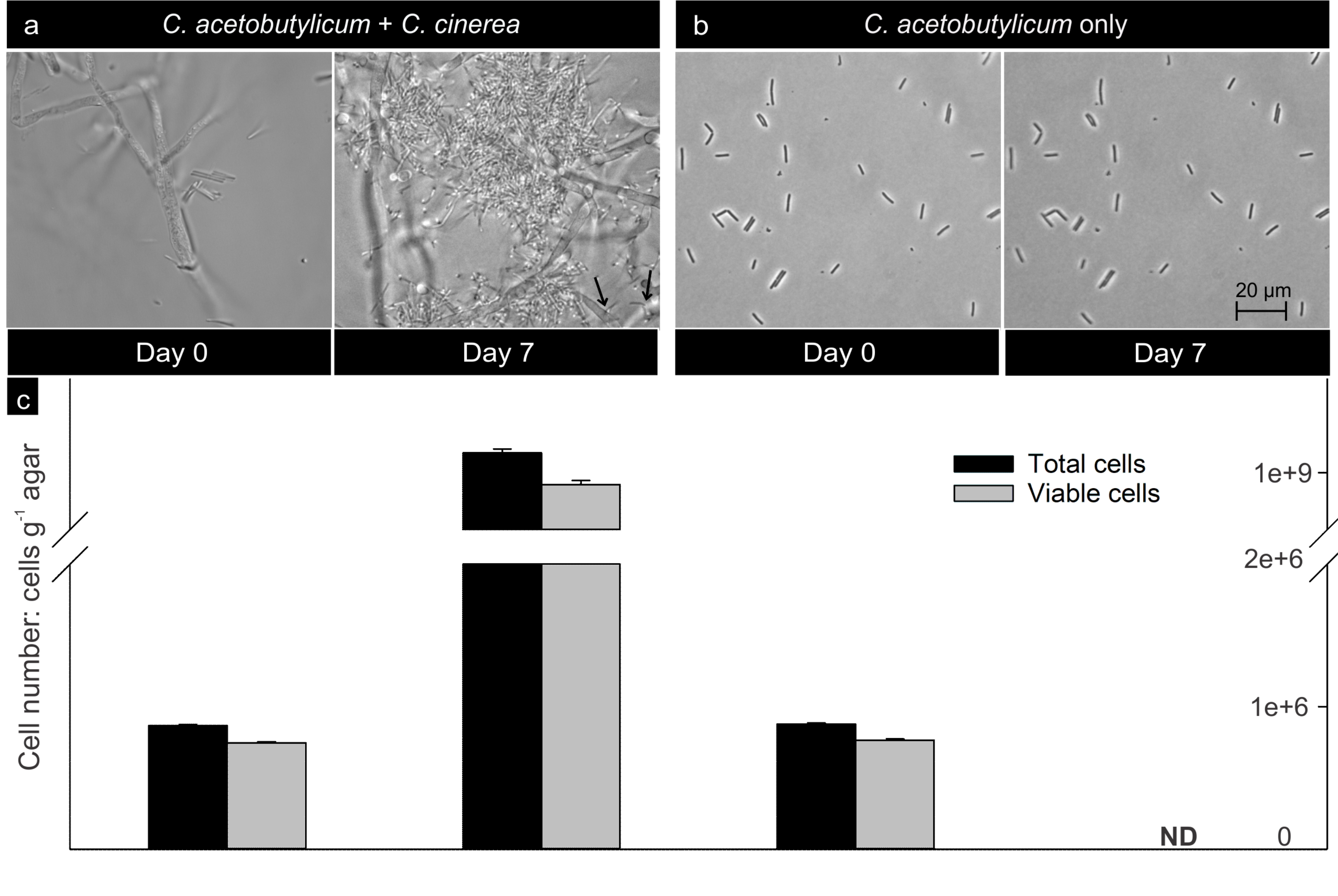

Supplement: FIG S1 [file mbio.00769-22-s0003.tif]

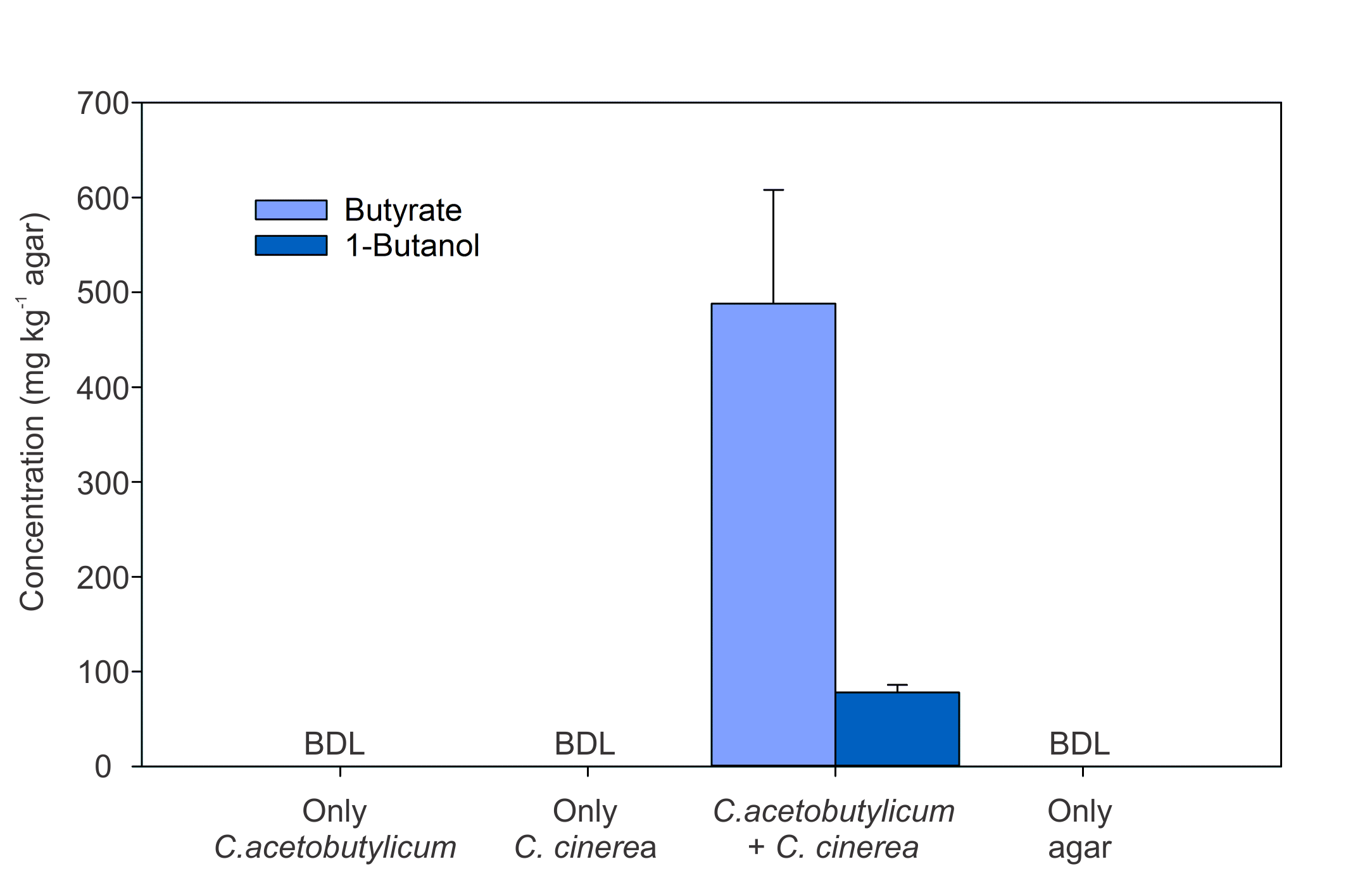

Supplement: FIG S2 [file mbio.00769-22-s0004.tif]

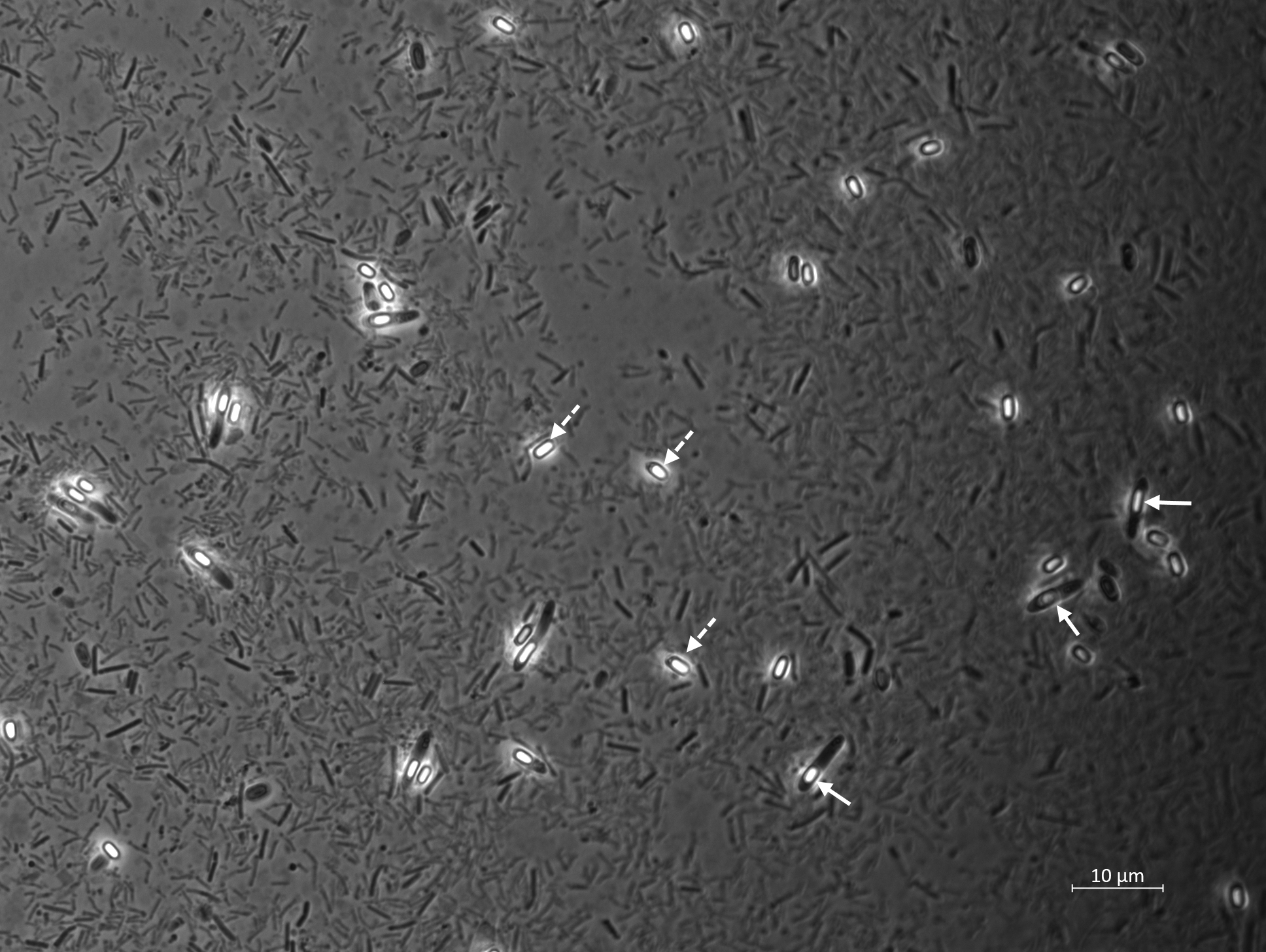

Supplement: FIG S3 [file mbio.00769-22-s0005.tif]

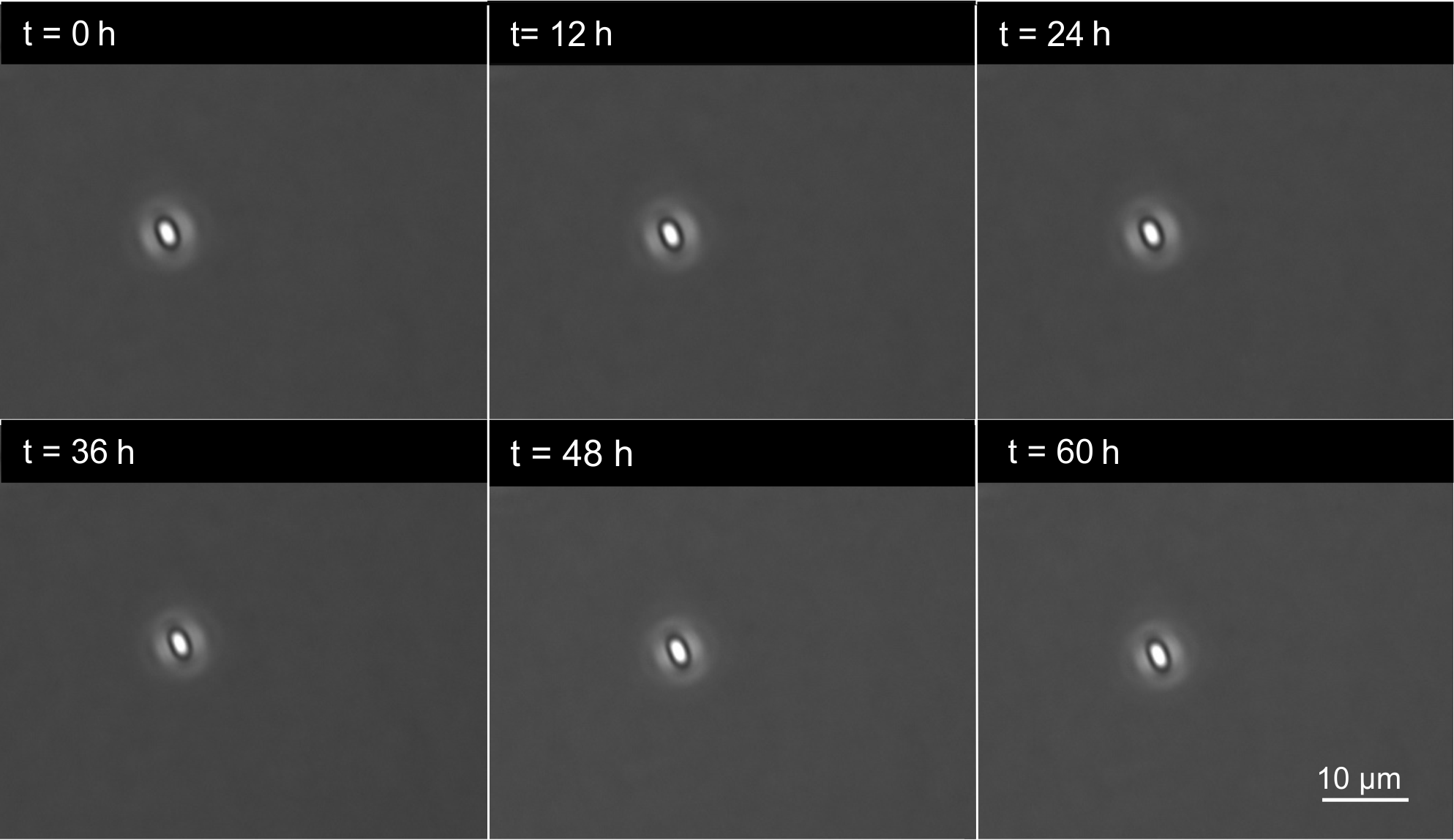

Supplement: FIG S4 [file mbio.00769-22-s0006.tif]

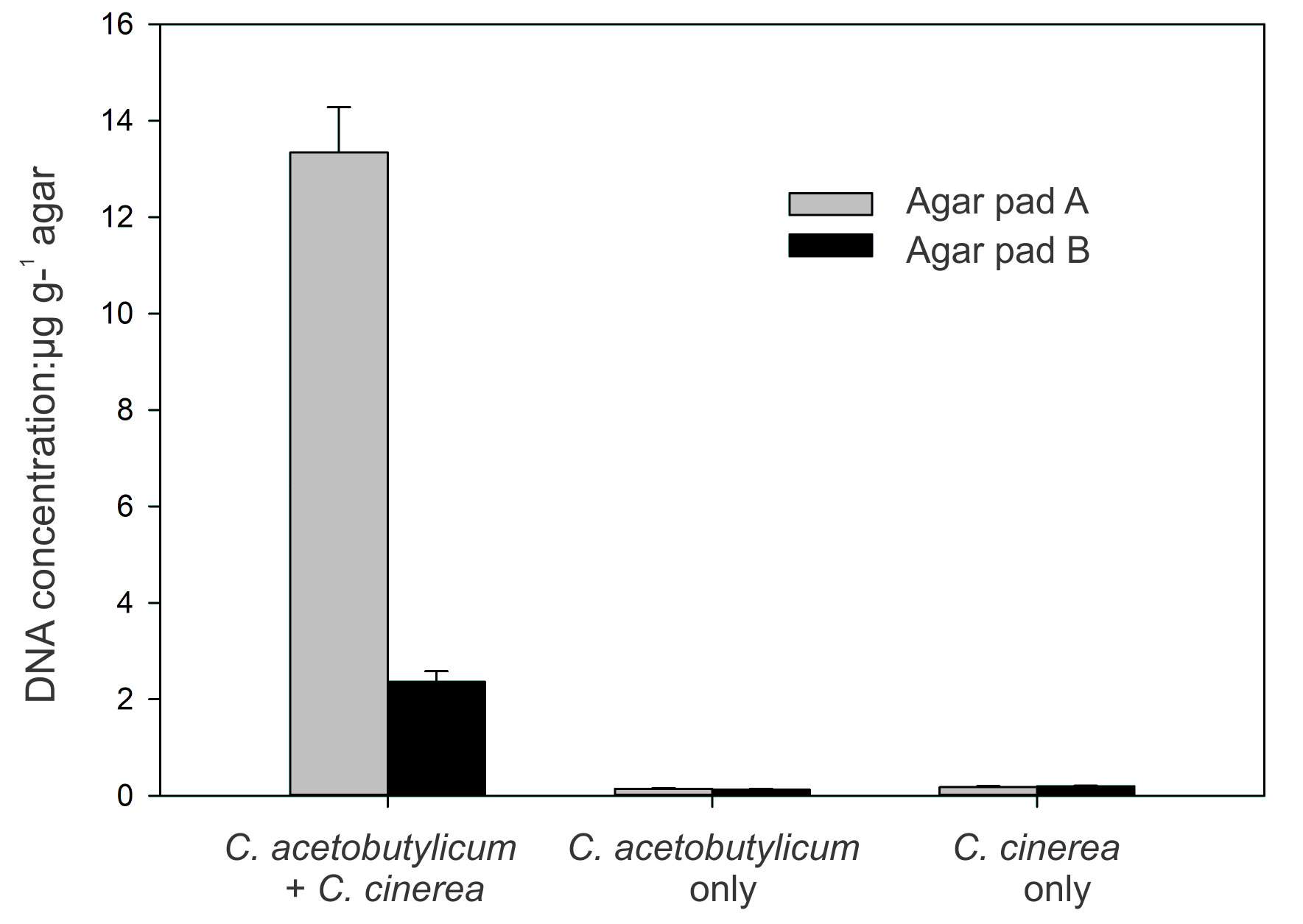

Supplement: FIG S5 [file mbio.00769-22-s0007.tif]
