# Supplementary material for: Impact of Fungal Hyphae on Growth and Dispersal of Obligate Anaerobic Bacteria in Aerated Habitats
Source: mBio. 2022 May 31;13(3):e00769-22. doi: 10.1128/mbio.00769-22 (PMC9239063; doi:10.1128/mbio.00769-22)
Supplement: TABLE S1 [file mbio.00769-22-s0002.pdf]

Table S1. Fraction of actively swimming *C. acetobutylicum* cells in liquid SM824 medium at different oxygen levels. The fraction refers to the % of *n* = 249-281 cells detected in the focal plane during microscopic observation of triplicate samples.

**Time of exposure** (min)	**Percentage of actively swimming cells**	
	80% air saturation	100% air saturation
~1	2.1 ± 0.2%	0.0 ± 0.0%
5	1.6 ± 0.2%	0.0 ± 0.0%
10	1.0 ± 0.2%	0.0 ± 0.0%
15	1.0 ± 0.2%	0.0 ± 0.0%
20	0.9 ± 0.2%	0.0 ± 0.0%
30	0.8 ± 0.0%	0.0 ± 0.0%
